# Human immunodeficiency virus-associated tuberculosis care in Botswana: evidence from a real-world setting

**DOI:** 10.1186/s12879-019-4401-9

**Published:** 2019-09-02

**Authors:** Ley Muyaya Muyaya, Esperance Manwana Musanda, Jacques Lukenze Tamuzi

**Affiliations:** 1 Palapye District Health Management Team, Department of Clinical services, Ministry of Health and wellness, PO Box 31, Palapye, 267 Botswana; 2 Department of Clinical services, Princess Marina Hospital, Ministry of Health and wellness, PO Box 258, Gaborone, 267 Botswana; 3 Otavi Hospital, Ministry of Health and Social, Private Bag 1005, Otavi, 264 Namibia

**Keywords:** Deaths, AIDS, Outcomes, Mycobacterium infection

## Abstract

**Background:**

Tuberculosis (TB) is among the world’s top public health challenges and the leading killer of people with HIV, yet is a treatable disease. This study aimed to assess, in a real-world setting, the implementation of antiretroviral therapy (ART) and Cotrimoxazole preventive therapy (CPT) policy, specific interventions proven to benefit patients in HIV-associated TB care.

**Methods:**

This retrospective cohort study was conducted in Botswana in the Serowe/Palapye district, a largely urban district with a high burden of HIV-associated TB with a high case fatality, at Segkoma and Palapye hospitals and their feeder clinics. Between 1 January 2013 and 31 December 2013, confirmed HIV-positive patients aged ≥15 years with a confirmed TB diagnosis and medical record available were included in the analysis. The Kaplan-Meier method was used to compare time to death for the group of patients on ART and the group of patients not on ART during TB treatment. Cox proportional hazard regression was undertaken to identify predictors of mortality.

**Results:**

Of the 300 patients included in the study, 217 (72%) were ART experienced at TB diagnosis. Of these, 86 (40%) had TB within 3 months following ART initiation. Of the 83 (28%) patients who were ART-naïve at TB diagnosis, 40 (48%) were commenced on ART during TB treatment, with 24 (60%) patients commencing within 4 weeks following TB treatment initiation. The overall ART uptake was 84%, while cotrimoxazole preventive therapy uptake was 100%. There were 45 deaths (15%), ART-experienced patients during TB treatment accounted for 30 deaths (30/257; 14%), while those who were not ART-experienced during TB treatment accounted for 15 deaths (15/43; 35%). There was a significant difference in survival time between patients with no ART use during TB treatment and those with ART use during TB treatment (log rank *p* < 0.001). Patients with no ART use during TB treatment were more likely to die within the first 2 months.

**Conclusion:**

The implementation of CPT policy is a substantial success. Strengthening the implementation of ART policy could improve survival among HIV-associated TB patients.

## Background

Tuberculosis (TB) is among the world’s top public health challenges and the leading killer of people with HIV, yet is a treatable disease [1, 2]. Globally, in 2017, there were an estimated 10 million cases of TB, and of these 10% were co-infected with HIV [2]. The number of deaths from TB in HIV-negative patients was 1.3 million, while deaths of HIV-positive patients numbered 300,000. Sub-Saharan Africa accounts for 84% of these TB-HIV co-infection deaths [2].

One of the targets of the Sustainable Development Goals for 2030 is to end the global TB epidemic. The End TB Strategy of the World Health Organization (WHO) calls for a 90% reduction in the TB mortality rate and an 80% reduction in the TB incidence rate by 2030, compared to rates for 2015 [3].

Over the past number of years, a large amount of evidence has been gathered about the negative impact of HIV on TB control [4, 5]. The HIV infection has markedly increased morbidity and mortality of Tuberculosis [4, 5]. Similarly, interventions to improve HIV-associated TB care have been found, and these form important components of the international standard of care for TB [6].

In a patient co-infected with TB and HIV, the two infections potentiate one another. The onset of tuberculosis in HIV-infected patient could result in the worsening of immunological functions with an increased risk of progression from HIV infection to AIDS and death [7]. Moreover, in an HIV high-burden setting, HIV infection has been found to be the most significant risk factor for developing active TB, and also the risk factor of TB reactivation for a person with latent TB [5]. Furthermore, the tremendously high frequency of undiagnosed disseminated TB in post-mortem studies of HIV/AIDS patients in the sub-Saharan Africa indicates that HIV-associated TB mortality is very likely considerably underestimated [8].

On the other hand, evidence from randomized control trials has shown that in patients with HIV-associated TB, the initiation of antiretroviral therapy (ART) during TB treatment could reduce mortality risk by between 64 and 95% [9]. In addition, the use of cotrimoxazole preventive therapy (CPT) among patients with HIV-associated TB reduced mortality by between 35 and 46% [10, 11].

Nevertheless, despite the evidence about ART and Cotrimoxazole, specific interventions that are proven to benefit patients, mortality in patients with HIV-associated TB is still high in sub-Saharan Africa [2, 12, 13], Thirty-five per cent of deaths among HIV-positive people are directly due to TB infection [2].

In Botswana, as a consequence of the high HIV burden, TB was declared as public health emergency in 2005. Although the incidence of TB is still among the highest in the world (300 per 100,000 in 2017) [2], there has been a sustained decline in the number of TB cases since universal access to ART was introduced in 2001 [14]. However, mortality in patients with HIV-associated TB is almost double of HIV-negative TB patients [2]. Therefore, there remains an urgent need to investigate the TB-HIV co-infection dynamics and determinants of these poor outcomes in a local and context-specific setting to inform care and policy.

In view of this poor prognosis in patients with HIV-associated TB, in 2012 prior the study period, Botswana had adopted the revised WHO (2010) guidelines which recommend that all patients with HIV-associated TB should be initiated on ART regardless of CD4 count, and also the use of cotrimoxazole preventive therapy (CPT) [15].

In this study on HIV-associated TB care in Botswana, we assessed in a real-world setting the survival of patients on treatment for HIV-associated TB and the implementation by healthcare providers of ART and CPT policy, specific interventions that are proven to benefit patients.

## Methods

### Study design

This was a retrospective cohort study using medical record review to assess the implementation of ART and cotrimoxazole policy, interventions proven to improve the care of adult patients with HIV-associated TB.

### Study setting

This retrospective cohort study was conducted in Botswana in the Serowe/Palapye district, a largely urban district with a high burden of HIV-associated TB with a high case fatality, at Segkoma and Palapye hospitals and their feeder clinics. In accordance with country policy, all patients presenting with TB are screened for HIV using the double HIV rapid test. During the study period, all patients with HIV-associated TB were eligible for ART regardless of their CD4 count. Patients with a CD4 count ≤100 cells/mm^3^ ART were to be started as soon as they were tolerating TB Treatment and patients with CD4 count > 100 cells/mm^3^ were to be started within 8 weeks and at least by the end of the initial phase of TB treatment. For those HIV patients without TB, they were eligible for ART if CD4 count < 350 or WHO clinical stage 3 or 4. While CPT was recommended for all patients with HIV-associated TB, Isoniazid preventive therapy (IPT) was not recommended for adults [15].

Generally, during the study period available investigations for TB included smear microscopy, chest radiology, abdominal ultrasonography, and fine-needle aspiration of lymph nodes for acid fast bacilli microscopy, which were done at primary and district hospital laboratories. Whilst culture and drugs sensitivity were only done at the National health laboratory but not on routine basis [15].

The standard first-line TB treatment during the study period was rifampicin, pyrazinamide, ethambutol and isoniazid during the two initial months (‘intensive phase’), followed by rifampicin, isoniazid and ethambutol for the next 4 months (‘continuation phase’). While, in patients with central nervous system diseases or TB infection in their bones, the continuation phase was extended to 12 months, in repeat TB cases the intensive was extended for 1 month with streptomycin included for the first 2 months and the continuation phase was extended to 5 months [15].

Furthermore, the standard ART first-line regimen for TB patients was: tenofovir (TDF) + emtricitabine (FTC) or Lamivudine (3TC) + efavirenz (EFV). Nevirapine (NVP) was used in cases of EFV intolerance. The standard ART second-line regimen was zidovudine (AZT) + lamivudine (3TC) and double dosed LPV/r (ritonavir-boosted lopinavir) [15].

At the primary healthcare level, TB and HIV services are co-localised under the same roof, while at hospital level these services are located on the same premises, but in different departments. Moreover, while the provision of ART prescription was only done at the referral clinic and hospital, TB treatment was provided at all the facilities where the patients were seen on an outpatient basis. Directly observed treatment was uniformly performed at all facilities.

### Study population

Between 1 January 2013 and 31 December 2013, confirmed [15] HIV-positive patients aged ≥15 years with a confirmed TB diagnosis by either laboratory or X-ray and medical records available were included in the analysis.

### Outcomes

The primary outcome was mortality, defined as any death that happened during TB treatment, which ranged from 6 months in new TB cases, 8 months in repeat TB cases, 12 months in TB meningitis cases, and up to 18 months in bone TB cases [6].

We defined ART uptake among HIV-associated TB patients during TB treatment as the commencement of ART during TB treatment or patients had been on ART prior TB diagnosis. ART experienced patients at the time of diagnosis were defined as HIV-associated TB patients who had been on ART prior TB diagnosis. Additionally, Major side effects were defined as any side effect that happened during TB treatment requiring first-line TB treatment to be discontinued [15].

Furthermore, we defined unexplained anaemia as anaemia with haemoglobin Hb < 10 g/dl with no anaemia etiology discovered after a comprehensive evaluation of the patient, and finally the Cotrimoxazole uptake among HIV-associated TB patients was defined as the administration of Cotrimoxazole preventive therapy during TB treatment.

### Data collection and statistical analysis

We reviewed medical records and TB and ART information management systems at the time of TB diagnosis and throughout the duration of the treatment to collect baseline demographic, clinical, pharmaceutical and laboratory data. The information gathered was used to complete a standard data-collection form. All HIV-associated TB patients with a medical record were included in this study.

The distribution of baseline characteristics was compared using chi-square and Fisher’s exact test for categorical variables and T-test for continuous variables. The Kaplan-Meier method was used to compare time to death for the group of patients on ART and the group of patients not on ART during TB treatment. Patients who did not experience the event measured were considered censored at the date of their last visit at the clinic while on treatment. To identify predictors of mortality, Cox proportional hazard regression was undertaken, with ART use during TB treatment, major side-effects, and other opportunistic infections included as time up-dated variables. We have used the Forward Stepwise (Likelihood Ratio) method to determine the final multivariate models. For the variables to be considered for inclusion in all multivariate models, we have used a univariate threshold of *p* ≤ .1. All results were presented in the form of hazard ratios (HR) and 95% confidence intervals (CI). The level of significance was set at .05.. Analyses were conducted using SPSS 21, IBM Corp., Armonk, New York.

### Ethics approval

Permission to conduct this study was obtained from Stellenbosch University (reference number S15/05/116), the Ministry of Health of Botswana (reference number PPME13/18/1 PS V (357)) and the local health authorities of the Serowe/Palapye district. Routine data regularly collected by healthcare providers for clinical care were used in this study. Therefore, informed consent was waived by the ethics committees, as the study used data previously collected for clinical routine care.

## Results

Between 1 January 2013 and 31 December 2013, 551 TB patients were initiated on TB treatment in the Serowe/Palapye district, 388 (70%) of them were co-infected with HIV. Of these, 300 (77%) were included in the study. However, 88 patients (23%) were excluded, as their medical records were missing or not captured.

The mean age of the overall group was 40 (Standard deviation [SD] 11) years. The majority of patients were from Botswana (99%), with more male (57%) than female patients (43%). Furthermore, more than half of the patients (69%) were not working and nearly all were single (85%) (Table 1).

**Table 1:** Baseline and follow up demographic and clinical characteristics of HIV-associated TB patients and comparison of mortality, Serowe/Palapye district Botswana 2013

Variables	Total *N* = 300 N (%)	Died Total:45 N (%)	Survivors Total:255 N (%)	P
Age group, year				
< 35	104 (35)	15 (33)	89 (35)	–
35–59	179 (60)	23 (51)	156 (61)	–
≥ 60	17 (6)	7 (16)	10 (4)	0.007
Male sex	170 (57)	28 (62)	142 (56)	0.514
Unemployed^a^	207 (69)	34 (76)	173 (68)	0.323
Smoker^b^	6 (2)	3 (7)	3 (1)	0.008
Body weight^c^ < 50 kg	102 (34)	17 (38)	85 (33)	0.023
Smear positive PTB	147 (49)	15 (33)	132 (52)	0.068
Extrapulmonary	80 (27)	13 (29)	67 (26)	0.861
Previous TB	34 (11)	4 (9)	30 (12)	0.799
Fever^d^	127 (42)	23 (51)	104 (41)	0.323
Productive cough^d^	212 (71)	31 (69)	181 (71)	0.863
Night sweats^d^	92 (30.7)	9 (20)	83 (33)	0.238
Loss of weight^d^	180 (60)	33 (73)	147 (58)	0.069
Duration of symptoms > 3 months^d^	44 (15)	7 (16)	37 (15)	0.509
No ART use during TB treatment	43 (14)	15 (33)	28 (11)	< 0.001
ART naïve	83 (28)	18 (40)	65 (26)	0.045
OI other than TB	19 (6)	12 (27)	7 (3)	< 0.001
CD4 counts cells/μl at TB^e^ < 200	111 (37)	21 (47)	90 (35)	0.086
Hemoglobin^f^ < 10 g/dl	85 (28)	22 (49)	63 (25)	0.004
Major Side effect	9 (3)	5 (11)	4 (2)	0.003
Diabetes mellitus	5 (2)	0	5 (2)	1
Cotrimoxazole prophylaxis	300 (100)	45 (100)	255 (100)	1

*TB* tuberculosis, *ART* antiretroviral therapy, *IRIS* immune reconstitution inflammatory syndrome;

Major side effect which were found included Hepatoxicity (8/9), and Stevens Johnson syndrome 1/9 OI: opportunistic infection

^a^ missing data for 22 patients (7.3%)

^b^ missing data for 125 patients (41.7%)

^c^ missing data for 87 patients (29%)

^d^ missing data for 21 to 25 patients (7 to 8.3%)

^e^missing data for 57 (19%)

^f^ missing data for 84 patients (28%)

**Fig. 1:**
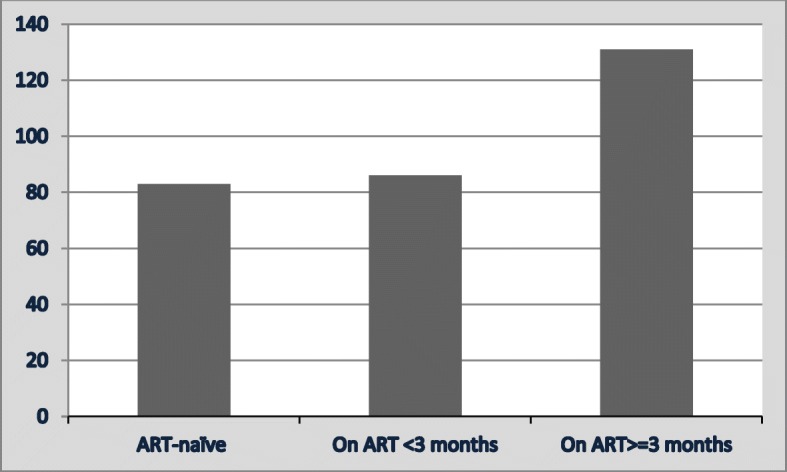
HIV-associated TB patients in subgroups defined according to the baseline status of ART use at the time of their TB diagnosis in Serowe/Palapye district Botswana 2013. The 3 subgroups of patients were categorised based on their ART status at the time of their TB diagnosis: 1) ART naïve: not on ART at TB diagnosis, 2) On ART> = 3 months: had been on ART for > = 3 months prior to their TB diagnosis, 3) On ART< 3 months: had been on ART for < 3 months at the time of their TB diagnosis. ART: antiretroviral therapy

Of the 300 patients with HIV-associated TB included in the study, 217 (217/300; 72%) were ART-experienced at the time of TB diagnosis. Of these, 86 (86/217; 40%) had TB within 3 months of ART initiation (Fig. 1). The remaining 83 (83/300; 28%) patients were ART-naïve at the time of TB diagnosis. Of these, 40 (40/83; 48%) were commenced on ART during TB treatment, with 24 (24/40; 60%) patients commencing within 4 weeks following TB treatment initiation. The overall ART uptake was 84% (257/300); in addition, the median time from ART initiation to TB diagnosis in ART experienced HIV-associated TB patients who had been on ART for ≥3 months at the time of their TB diagnosis was 37 months (interquartile range [IQR]: 13.93–75.97). Moreover, CPT was given to every patient included in the study.

There were 45 deaths (45/300; 15%) during the study period. ART-experienced patients during TB treatment accounted for 30 deaths (30/257; 14%), with 18 deaths (18/131; 14%) amongst those who had been on ART for ≥3 months at the time of their TB diagnosis and 12 deaths (12/126; 10%) amongst those with recent ART initiation (had TB within 3 months of ART initiation or initiated on ART following TB treatment). While those who were not ART-experienced during TB treatment accounted for 15 deaths (15/43; 35%).Eight patients (3%) defaulted treatment and five patients (2%) were transferred to other facilities.

At baseline and during follow up, there were a number of significant differences between patients who died and those who survived. Those who died were more likely to be older (*p* = 0.007), a smoker (7% vs. 1%; *p* = 0.008), had a body weight < 50 kg (38% vs. 33%; *p* = 0.023), had unexplained anaemia (Haemoglobin (Hb) < 10 g/dl) (49% vs. 25%; *p* = 0.004), had an opportunistic infection other than TB (27% vs. 3%; *p* = 0.001) and received no ART during TB treatment (33% vs. 11%; *p* < 0.001). Furthermore, they were more likely to have developed a major side effect (11% vs. 2%; *p* = 0.003) (Table 1).

In the multivariate analysis (Table 2), no ART use during TB treatment (*p* < 0.001), opportunistic infections other than TB (p < 0.001), age ≥ 60 years (*p* = .002), haemoglobin < 10 g/dl (*p* = 0.029) and major side effects (*p* = 0.007) were significantly associated with higher mortality.

**Table 2:** Summary of Predictors of mortality in HIV-associated TB patients in Serowe/Palapye district Botswana 2013

Variables	Hazard ratio (95% CI)	P	Adjusted HR (95% CI)	P
Age group, year				
< 35	1	–	–	–
35–59	0.89 (0.46–1.7)	0.722	1.5 (0.75–3)	0.245
≥ 60	3.15 (1.28–7.73)	0.012	4.8 (1.8–13)	0.002
Male sex	1.22 (0.67–2.23)	0.513	–	–
Unemployed	1.2 (0.59–2.42)	0.627	–	–
Smoker	2.53 (0.76–8.42)	0.130	0.66 (0.13–3.4)	0.619
Body weight < 50 kg	2.1 (0.94–4.71)	0.072	1.8 (0.7–4.5)	0.205
Smear positive PTB	0.5 (0.27–0.93)	0.028	–	–
Extrapulmonary	1.12 (0.59–2.13)	0.739	–	–
Previous TB	0.74 (0.27–2.01)	0.571	–	–
Fever	1.58 (0.85–2.94)	0.153	–	–
Productive cough	0.94 (0.46–1.92)	0.870	–	–
Night sweats	0.55 (0.26–1.16)	0.116	–	–
Loss of weight	2.33 (1.07–5.04)	0.032	–	–
Duration of symptoms > 3 months	3.85 (0.47–31)	0.207	–	–
No ART use during TB treatment	2.49 (1.31–4.73)	0.005	5.6 (2.9–11)	< 0.001
ART naïve	1.2 (0.66–2.2)	0.569	–	–
OI other than TB	7.76 (3.96–15.25)	< 0.001	8.5 (4–18.4)	< 0.001
CD4 counts cells/μl at TB < 200	2.02 (1.01–4.04)	0.046	–	–
Hemoglobin < 10 g/dl	2.4 (1.3–4.5)	0.007	2.44 (1.3–4.6)	0.029
Major Side effect	4.84 (2–12.3)	0.001	5 (1.6–17)	0.007
IRIS	1.1 (0.53–2.27)	0.808	–	–
Diabetes mellitus	0.049 (0–2559)	0.586	–	–

*TB* tuberculosis, *ART* antiretroviral therapy, *IRIS* immune reconstitution inflammatory syndrome, *HR* hazard ratio, *CI* Confidence Interval. Major side effect found were Hepatoxicity (8/9), and Stevens Johnson syndrome 1/9 OI: opportunistic infection, and ART use during TB treatment: time-updated variables

**Fig. 2:**
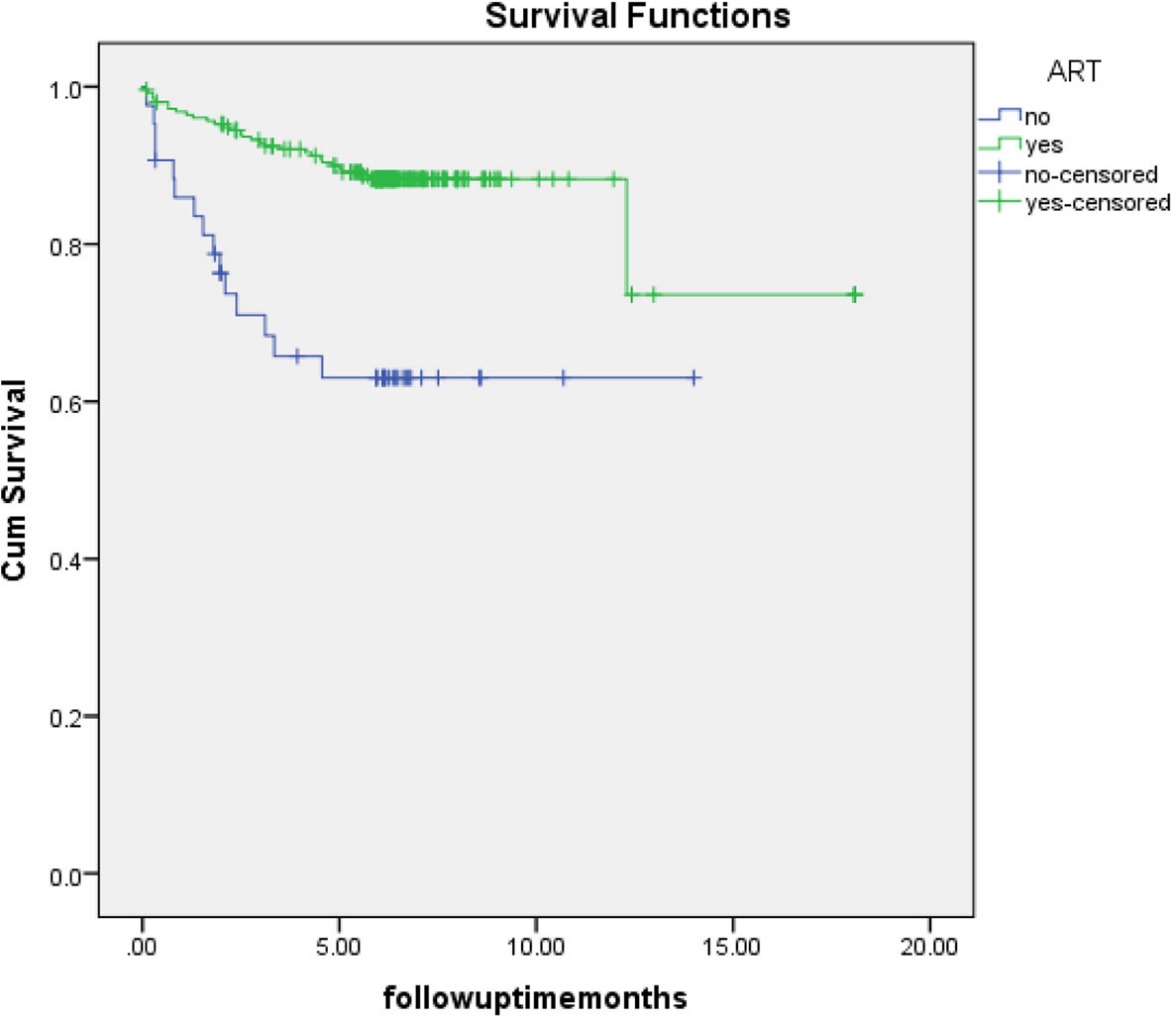
Kaplan–Meier survival estimates according to ART use during TB treatment. The 2 subgroups of patients were categorised based on the use of ART during the TB treatment: 1) yes ART: commenced on ART during TB treatment or had been on ART prior to their TB diagnosis, 2) no ART: ART naïve not commenced on ART during TB treatment. ART: antiretroviral therapy

There was a significant difference in survival time between the group of patients with no ART use during TB treatment and those with ART use during TB treatment (log rank p < 0.001). The survival curves illustrates that most patients died in the first 5 months; there were few deaths after 5 months. However, patients with no ART use during TB treatment were more likely to die within the first 2 months (Fig. 2).

## Discussion

Our study showed that the burden of HIV-associated TB and related mortality is still high. There is a substantial success in the implementation of Cotrimoxazole preventive therapy with an uptake of 100%, however there are still gaps in the implementation of ART policy for HIV-associated TB patients despite the national policies, strategies and guidelines.

Among HIV-associated TB patients who were ART-experienced at the time of TB diagnosis, 40% had TB within 3 months following ART initiation a large proportion that is similar to findings reported in other studies [16–19]. These findings highlight the need that known strategies for stopping the spread of TB should be strengthened, prioritised, implemented and scaled up in this setting. These strategies include intensified case finding aimed at prompt diagnosis of TB in ART service, both at baseline and during follow-up visits, thereby potentially reducing morbidity, mortality and TB transmission [6]. Studies have reported that effective baseline screening reduces the risk of ‘unmasking’ TB-immune reconstitution disease following ART initiation [20, 21], which can sometimes be life-threatening [22, 23].

The 4-symptom (current cough, night sweats, weight loss, fever) based screening tool integrated in the WHO guidelines for intensified TB case finding has poor specificity and sub-optimal sensitivity, a meta-analysis of around 10,000 HIV positive patients found that reporting at least one of four symptom had an overall sensitivity of 79% and a specificity of 50% during active screening for TB [24]. Therefore, due to poor specificity a large numbers of identified patients may need further diagnostic evaluation. Moreover, the sub-optimal sensitivity means that 10 - 20% of asymptomatic patients with active TB are missed. Furthermore, The Xpert MTB/RIF assay substantially increased TB case finding compared to smear microscopy, diagnoses all smear-positive cases and around 40–70% smear-negative culture-positive cases with a very high specificity, fast TB diagnosis and detection of rifampicin resistance [25]. In addition, the lateral flow urine lipoarabinomannan assay (LF-LAM) may be used to assist in the diagnosis of TB in HIV positive adult in-patients with signs and symptoms of TB who have a CD4 count ≤100 cells/μL, or HIV positive patients who are seriously ill regardless of CD4 count or with unknown CD4 count [26]. (Seriously ill is defined based on 4 danger signs: respiratory rate > 30/min, temperature > 39 °C, heart rate > 120/min and unable to walk unaided) [26]. Moreover, the screening of all HIV positive in-patients using lateral flow urine-LAM assay could substantially reduce the risk of being discharged from hospital with undiagnosed TB [27].

Additionally, it has been documented that in the first few months following ART initiation, a large proportion of the TB burden is due to ‘unmasking’ asymptomatic or minimally symptomatic disease that was present at baseline but missed during pre-ART screening [18, 20]. Therefore, in countries with a high HIV-associated TB burden, such as Botswana, there is a strong argument for microbiological and X-ray pre-ART screening of all patients, regardless of symptoms [25]. In addition, because TB risk is strongly related to incomplete or poor immune recovery [18], serial screening might best be targeted in those starting ART or those with poor immune recovery [25].

In this cohort of HIV-associated TB patients, of the 217 (72%) patients who were ART-experienced at the time of TB diagnosis, 60% patients had been on ART for more than 3 month at the time of their TB diagnosis, with median time from ART initiation to TB diagnosis of 37 months (interquartile range [IQR]: 13.93–75.97). Our finding suggest that further interventions to prevent TB among people living with HIV, such as IPT and intensified case finding, are warranted in this setting. Moreover, studies conducted in Brazil and South Africa report that whereas IPT and ART were both effective in reducing TB risk among adult people living with HIV, the combination of the two interventions was more protective than either alone, resulting in an 80 and 89%, respectively, reduction in TB rates when compared to patients receiving neither intervention [28, 29].

Among HIV-associated TB patients who were ART-naïve at the time of TB diagnosis in this study, only 48% were commenced with ART during TB treatment – a proportion below that reported by other studies [30, 31]. Possible reasons might be poor implementation of clinical guidelines, the challenges of integrating TB-HIV care, and inadequate clinical monitoring [30]. These findings highlight the gap in the delivery of HIV-associated care in Botswana despite national policies, strategies and guidelines and the overwhelming evidence base of the benefit conferred by early initiation of ART and integration of ART and TB treatment [32–34].

Moreover, our study showed that mortality was 15% in HIV-associated TB patients in Botswana, which is still high despite the ART and CPT uptake of 84 and 100%, respectively. A similar proportion of mortality was reported in Swaziland and Malawi [12, 13]. However, the analysis of the characteristics of this cohort of HIV-associated TB patients showed that patients who died were more likely to have other co-morbidities such as other opportunistic infections, anaemia, and not commencing with ART. ART has been documented as reducing mortality risk by between 64 and 95% [25, 35]. The contribution of other opportunistic infections such as cryptococcal disease, cytomegalovirus and bacterial infections to mortality in patients with HIV-associated TB has been documented [25]. Therefore, the optimised case management for HIV-associated TB could include baseline and serial screening of other opportunistic infections using improved diagnostic tools such as blood cultures, cryptococcal antigen screening and full blood count, as well as the early initiation of ART, treatment options for co-infection [25] and close clinical monitoring of side effects.

The findings of this study suggest that in Botswana while there had been a substantial success in the implementation of Cotrimoxazole preventive, there is a still a gap in the implementation of ART policy on the ground by clinical care providers. Although some progress has been made by the country in adopting (in 2016) the Treat All Strategy for all people living with HIV, with initiation on ART regardless of CD4 level, the introduction of Cryptococcal antigen screening for HIV patients with CD4 count ≤100 cells/mm^3^ and the new TB diagnostic tool,The Xpert MTB/RIF assay (Cepheid Inc., Sunnyvale, CA, USA) [36]. To address the HIV-associated TB epidemic, we need intrepid but responsible action, without which the future will merely mirror the past. Consequently, there is an urgent need to redouble efforts to implement the WHO-endorsed strategies to prevent TB as a package, which include intensified case finding in pre-ART and follow-up screening in ART services, infection control, IPT and the early initiation of ART [3, 6]. In addition, integrated care at the facility level for patients with HIV-associated TB has also been shown to improve treatment outcomes for both TB and HIV [25].

One of the strengths of this study was the use of data from routine medical records; therefore it is representative of what is happening on the ground. The limitations of this study included the fact that it was a retrospective observational study, using data from routine medical records in the public health sector, and sometimes data were missing or inconsistent; For example, CD4 count was not stratified among ART naïve patients to analyze the relation with timing of ART initiation and mortality as so much data were missing. In addition, given the inclusion of different forms of TB as well as the wide range of times since ART initiation, heterogeneity was another limitation of this study. Furthermore, the lack of testing for drug resistance especially among TB repeat cases and data on ART adherence was also a limitation of this study.

Further research could be undertaken to understand the barriers on the path of HIV-associated TB care from patient and provider perspectives. Although this study was conducted in one district in Botswana, as this district is similar to other districts with regard to HIV-associated TB epidemiology, the results can be generalised to the country as a whole.

## Conclusion

We conclude that our study in Botswana, a setting with limited resources, there is a substantial success in the implementation of cotrimoxazole preventive therapy policy. However there is a still a gap in the implementation of ART policy on the ground by clinical care providers. Our findings suggest that strengthening the implementation of ART policy and optimising the HIV-associated TB case management could improve the survival among HIV-associated TB patients.

## Data Availability

The current study included human subjects therefore to avoid loss of confidentiality; the dataset of this study is not publicly available. The dataset may be available upon reasonable request to the corresponding author.

## Abbreviations

3TC: Lamivudine
3TC: Lamivudine
ART: Antiretroviral therapy
AZT: Zidovudine
CI: Confidence intervals
CPT: Cotrimoxazole preventive therapy
EFV: Efavirenz
FTC: Emtricitabine
Hb: Haemoglobin
HIV: Human immunodeficiency virus
HR: Hazard ratios
IPT: Isoniazid preventive therapy
IRIS: Immune reconstitution inflammatory syndrome
LF-LAM: Lateral flow urine lipoarabinomannan assay
LPV/r: Ritonavir-boosted lopinavir
NVP: Nevirapine
SD: Standard deviation
TB: Tuberculosis
TDF: Tenofovir
WHO: World health organisation
